# Heart in a Dish: From Traditional 2D Differentiation Protocols to Cardiac Organoids

**DOI:** 10.3389/fcell.2022.855966

**Published:** 2022-02-17

**Authors:** Gustavo Ramirez-Calderon, Giovanni Colombo, Carlos A. Hernandez-Bautista, Veronica Astro, Antonio Adamo

**Affiliations:** Biological and Environmental Science and Engineering Division, King Abdullah University of Science and Technology, Thuwal, Saudi Arabia

**Keywords:** organoids, cardiac differentiation, disease modeling, cardiogenesis, pluripotent stem cell (PSC), cardiac development, cardiac maturation

## Abstract

Human pluripotent stem cells (hPSCs) constitute a valuable model to study the complexity of early human cardiac development and investigate the molecular mechanisms involved in heart diseases. The differentiation of hPSCs into cardiac lineages *in vitro* can be achieved by traditional two-dimensional (2D) monolayer approaches or by adopting innovative three-dimensional (3D) cardiac organoid protocols. Human cardiac organoids (hCOs) are complex multicellular aggregates that faithfully recapitulate the cardiac tissue’s transcriptional, functional, and morphological features. In recent years, significant advances in the field have dramatically improved the robustness and efficiency of hCOs derivation and have promoted the application of hCOs for drug screening and heart disease modeling. This review surveys the current differentiation protocols, focusing on the most advanced 3D methods for deriving hCOs from hPSCs. Furthermore, we describe the potential applications of hCOs in the pharmaceutical and tissue bioengineering fields, including their usage to investigate the consequences of Severe Acute Respiratory Syndrome CoronaVirus 2 (SARS-CoV2) infection in the heart.

## Introduction

The knowledge on human cardiogenesis has been chiefly based on either post-mortem studies or animal models for a long time. Consequently, the use of human pluripotent stem cells (hPSCs) as an *in vitro* model to study cardiac development has flourished over the last two decades. hPSCs are suitable for regenerative medicine, tissue engineering, and drug screening applications owing to their unique proliferative capacity and potential to differentiate into virtually all somatic cell types.

Here, we emphasize the latest breakthroughs in the fields of cell biology and tissue bioengineering that open the road for the generation of 3D human cardiac organoids (hCOs).

## Cardiac Development at a Glance

**FIGURE 1:**
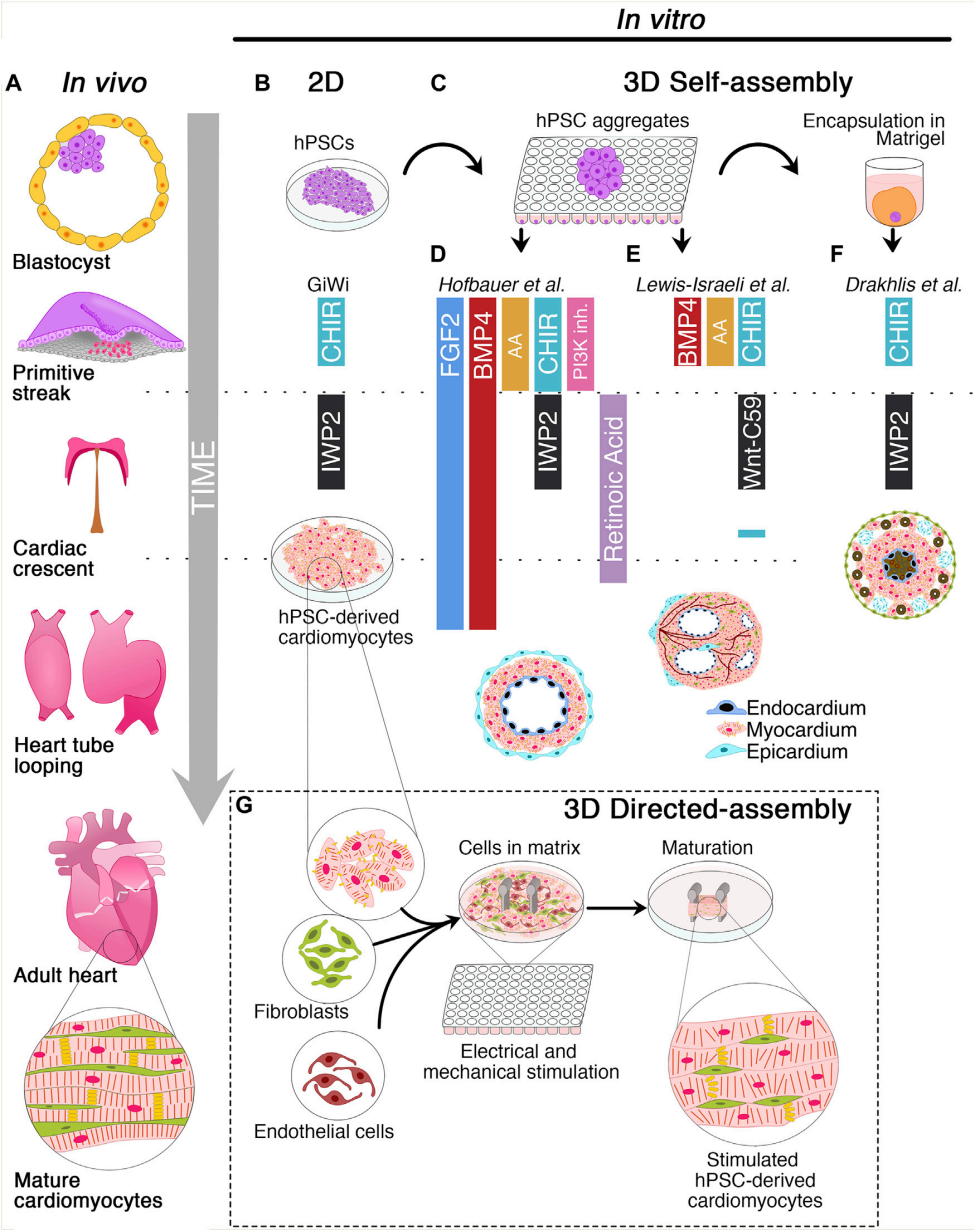
Schematic of cardiac development and 2D/3D culture methods for the derivation of human cardiac organoids. **(A)** Timeline of cardiac development *in vivo*. The blastocyst originates the primitive streak from which the mesodermal layer emerges. Cardiovascular progenitors migrate through the primitive streak to form the cardiac crescent at the embryo’s midline and generate the heart tube. Subsequently, heart tube looping and remodeling produce the final heart architecture. **(B)** Description of 2D GiWi differentiation protocol. hPSCs are treated with CHIR99021 (GSK3 inhibitor/Wnt activator) to induce primitive streak and mesoderm, followed by IWP2 (Wnt-inhibitor) treatment to trigger the cardiac commitment. The GiWi 2D protocol generates immature fetal-like hPSC-derived cardiomyocytes. **(C)** 3D self-assembly of human cardiac organoids begins with hPSCs aggregation in ultra-low attachment plates. However, only Drakhlis et al. used an additional hPSC aggregate encapsulation in Matrigel. **(D)** Hofbauer et al. implemented FGF2 and BMP4 throughout the mesoderm induction, cardiac mesoderm, and cardiomyocyte specification. Additionally, this method relies on Activin A (AA), CHIR99021, and LY294002 (PI3K- inhibitor) for mesoderm induction, IWP2, and retinoic acid for cardiac mesoderm induction. Additional co-culture with epicardial aggregates generates hCOs with chamber-like structures and three layers composed of epicardium, myocardium, and endocardium. **(E)** Lewis-Israeli et al. adopted a modified GiWi protocol by adding BMP4, AA, and CHIR99021 for mesodermal induction and Wnt-C59 (Wnt-inhibitor) for cardiac mesoderm formation. A subsequent CHIR99021 treatment triggers epicardial induction. These organoids develop chambers and vessel-like structures. **(F)** Drakhlis et al. described a GiWi protocol to generate hCOs with an inner and outer layer resembling the anterior and posterior foregut endoderm (brown color). These hCOs have a mid-myocardial layer without chamber-like structures and some epicardial-like cells (septum-transversum-like anlagen) in the outer layer. **(G)** Directed assembly of hCOs combines hPSC-derived cardiomyocytes with fibroblasts and endothelial cells in a hydrogel matrix surrounding two elastomeric poles. After hydrogel condensation, hCOs are mechanically or electrically stimulated or both to induce a mature-like phenotype.

The heart is the first organ to be formed during embryonic development to pump nutrients, hormones, proteins, and waste once the embryo’s size limits diffusion (Vincent and Buckingham, 2010). Heart development begins shortly after embryo gastrulation (Buckingham et al., 2005). During mesodermal commitment, the anterior primitive streak cells start expressing the master regulator of cardiac progenitor specification Mesoderm Posterior BHLH Transcription Factor 1 (*MESP-1*), together with the surface markers Kinase Insert Domain Receptor (*KDR*) and Platelet-Derived Growth Factor Receptor (*PDGFR*) and migrate bilaterally forming the cardiac crescent at the midline, the future heart’s site (Saga et al., 1996; Saga et al., 2000; Bondue et al., 2011; Lescroart et al., 2014; Burridge et al., 2015; Lescroart et al., 2018). As the embryo grows, the cardiac crescent fuses at the midline and forms the heart tube, which contains an inner layer of endocardial cells and an outer layer of myocardial cells and begins to pump blood almost immediately (Vincent and Buckingham, 2010; Burridge et al., 2015; Sahara et al., 2015). Independent of the crescent, a subset of cells from the proepicardial organ invades the developing heart tube to form the epicardium, the outermost layer of the heart. A further extension, looping, and remodeling of the cardiac tube, together with the contribution of the cardiac neural crest cells originating from the dorsal neural tube, leads to the valve and septum formation and the generation of ventricular and atrial chambers (Vincent and Buckingham, 2010; Burridge et al., 2015; Sahara et al., 2015) (Figure 1A). The developed heart consists of multiple cell types including cardiomyocytes (CMs), vascular smooth muscle cells, endothelial cells, fibroblasts, and conductive system cells. The spatiotemporal assembly of these cells generates the three-layer structure of the heart, composed of endocardium, myocardium, epicardium, and the interconnected four chambers of the organ. Intriguingly, CM maturation continues until adulthood (Yang et al., 2014). CMs undergo dynamic changes during cardiac development to reach the mature contractile force and electrophysiological phenotype, including variations in cell size, sarcomere ultrastructure, metabolic substrate, mitochondrial content, nuclei number, gap junction distribution, and proliferation rate (Yang et al., 2014).

## Molecular Cues Driving Human Pluripotent Stem Cells Differentiation Into Cardiomyocytes

Protocols for hPSC differentiation into CMs rely on three steps mimicking the sequential modulation of pathways driving cardiogenesis *in vivo* (Laflamme et al., 2007; Yang et al., 2008; Kattman et al., 2011). The first stage is based on the use of Activin A (AA), Bone Morphogenetic Protein 4 (BMP4), and basic Fibroblast Growth Factor (bFGF) to induce primitive streak and mesoderm specification. AA is a potent inducer of mesoderm and endoderm in *Xenopus* embryonic explants (Marvin et al., 2001). Additionally, the secretion of AA, Transforming Growth Factor-Beta (TGF-*β*), and BMP4 from hypoblast and anterior endoderm drives the cardiac induction in chick embryos (Smith and Eichele, 1991; Ladd et al., 1998; Nakajima et al., 2002; Laflamme et al., 2007). Since Wnt inhibition promotes heart formation in *Xenopus* (Marvin et al., 2001), chicks, and mouse embryonic stem cells (Ueno et al., 2007), the second stage relies on Wnt inhibitors, such as DKK-1, IWP2, IWP4, or Wnt-C59 to promote cardiac mesoderm differentiation. The third stage aims at developing cardiovascular lineages and promoting the specification of a non-homogenous population of CMs, smooth muscle, and endothelial cells (Yang et al., 2008; Kattman et al., 2011). The most employed chemically-defined protocol known as GiWi (GSK-inhibition/Wnt-inhibition) allows the generation of about 80% of CMs from several hPSC lines. This method drives the specification into mesoderm precursors by treating the cells with CHIR99021, a glycogen synthase kinase-3*β* (GSK-3*β*) inhibitor that activates the canonical Wnt/*β*-catenin pathway. Mesodermal precursors are then differentiated into cardiac mesodermal progenitors by Wnt inhibition (Lian et al., 2012; Lian et al., 2013; Burridge et al., 2014; Lian et al., 2015). Unfortunately, the planar architecture and the high heterogeneity of the hPSC-derived CMs hinder the application of 2D protocols for regenerative medicine purposes.

## 3D Culture Methods for the Derivation of Human Cardiac Organoids

Human organoids can be obtained through the self-assembly of hPSCs induced by morphogens or by the directed assembly from previously differentiated stem cells (Li and Izpisua Belmonte, 2019; Schutgens and Clevers, 2020; Zhao et al., 2021; Lewis-Israeli et al., 2021a) (Figures 1B–G). Although the derivation of human organoids of the intestine (Sato et al., 2011), brain (Lancaster et al., 2013), and lung (Miller et al., 2019) was reported several years ago, hCOs have been only recently generated, likely due to the developmental and structural complexity of the heart (Lewis-Israeli et al., 2021a; Zhao et al., 2021). hCOs resemble key embryo developmental features, such as primitive streak induction, cardiac specification, chamber formation by cavitation, epicardium formation, and vascularization. Moreover, hCOs are composed of diverse cell subtypes, such as CMs, endothelial, smooth muscle, and epicardial cells, including endodermal derivatives (Drakhlis et al., 2021a; Drakhlis et al., 2021b; Hofbauer et al., 2021; Lewis-Israeli et al., 2021b).

In the following sections, we will discuss the two most recent approaches to generate hCOs from hPSCs (Table 1). The first section will navigate through the “self-assembly” method that exploits the intrinsic property of hPSC aggregates to drive a spatiotemporal organized differentiation mediated by exogenous morphogens. The second part of the review will present “directed assembly” protocols that rely on the co-culture of hPSC-derived CMs, endothelial, or mural cells on different supports, such as extra cellular matrix (ECM), scaffolds, or bioengineering devices.

**TABLE 1:** Generation of hCOs by the self-assembly and the directed assembly methods.

References	Cell source	Method	Platform	Applications	hCO features
Drakhlis et al. (2021a)	hESCs and hiPSCs	Self-assembly	Round bottom ultra-low attachment 96-well plate, Matrigel-embedded	Modeling early cardiomyogenesis and genetic heart defects *in vitro* (NKX2.5-KO HFOs recapitulate *in vivo* phenotype)	HFOs including inner (anterior foregut endoderm-like), myocardial, and outer (posterior foregut endoderm-like) layers
Hofbauer et al. (2021)	hESCs and hiPSCs	Self-assembly	Round bottom ultra-low attachment 96-well plate, later co-culture with hPSC-derived epicardial clusters	Modeling early cardiomyogenesis (HAND1-KO hCOs) and developmental injury *in vitro* (cryoinjury of hCOs)	Cavity-containing hCOs, including endocardial and myocardial layer. Outer epicardial layer obtained by co-culture with hPSC-derived epicardial clusters
Lewis-Israeli et al. (2021a)	hESCs and hiPSCs	Self-assembly	Round bottom ultra-low attachment 96-well plate	Modeling early cardiomyogenesis and disease condition *in vitro* (pregestational diabetes-induced congenital heart defects)	Cavity-containing hCOs, including endocardial, myocardial and epicardial layers
Ma et al. (2015); Hoang et al. (2018); Hoang et al. (2021)	hESCs and hiPSCs	Self-assembly	Micropatterned 6-well plate	Developmental drug toxicity assays (pregnancy risk drugs)	hCOs include a central area of CMs with a perimeter of myofibroblasts
Tiburcy et al. (2017)	(hESCs and hiPSCs) hPSC derived-CMs and human foreskin fibroblasts	Directed assembly	Casting in medical-grade type I collagen hydrogels, later mechanical stimulation by dynamic stretching between two elastomeric pillars	Advance CMs maturation and modeling heart failure *in vitro* (chronic catecholamine overstimulation)	EHTs with fetal-like CMs
Ronaldson-Bouchard et al. (2018)	hiPSCs	Directed assembly	Casting in fibrin hydrogels, later mechanical stimulation by dynamic stretching between two elastomeric pillars and electrical stimulation	Improving hPSC-derived CM maturation and modeling pathological cardiac hypertrophy (via endothelin-1 treatment)	EHT with extensive T-tubule network and mature calcium handling
Mills et al. (2017); Mills et al. (2019); Mills et al. (2021)	hESCs and hiPSCs	Directed assembly	Heart-dyno: casting in collagen I and Matrigel hydrogels in a well insert containing two elastomeric pillars	Screening for cardiac maturation, cardiomyocyte proliferation, and drugs to treat SARS-CoV-2-induced cardiac dysfunction	hCOs with fatty acid oxidation metabolism, DNA damage response, and cell cycle arrest

## Self-Assembly Method

### Human Cardiac Organoids Generation in Ultra-Low Attachment Platforms

In recent years, the round bottom ULA plates have been used as a high-throughput platform to screen favorable conditions for hCO generation (Drakhlis et al., 2021a; Drakhlis et al., 2021b; Hofbauer et al., 2021; Lewis-Israeli et al., 2021b). Indeed, it has been shown that self-assembling hPSC-3D aggregates can give rise to hCOs, even in Matrigel-free protocols (Hofbauer et al., 2021; Lewis-Israeli et al., 2021b) (Figure 1C). Before inducing the differentiation, a defined number of hPSCs is seeded in each well, and the round bottom ULA plate is centrifuged to promote hPSC aggregate formation. From these aggregates, employing a modified GiWi protocol, it is possible to generate hCOs resembling crucial stages of early cardiomyogenesis, such as mesoderm specification, cardiac mesoderm induction, and cardiac cavity formation (Drakhlis et al., 2021a; Drakhlis et al., 2021b; Hofbauer et al., 2021; Lewis-Israeli et al., 2021b).

Hofbauer and others developed an ECM-free protocol to produce hPSC-derived hCOs forming beating chamber-like architectures with an inner endocardial and an outer myocardial layer (Figure 1D) (Hofbauer et al., 2021). This method exploits an optimized WNT and AA treatment during mesoderm induction and a reduced retinoic acid stimulation during the cardiac mesoderm stage to direct the differentiation towards a ventricular-like fate (Zhang et al., 2011). Interestingly, this model demonstrated that a WNT-BMP-HAND1 axis controls the generation of the chamber-like structures and that the cavitation process is independent of CM differentiation. Indeed, the disruption of the HAND1 signaling suppresses the chamber formation process but does not affect CM differentiation, indicating that cell-type specification and organ morphogenesis are independently regulated processes. Notably, when hPSC-derived epicardial aggregates are co-cultured with these hCOs, they recapitulate the canonical three-layer architecture composed of epicardium, myocardium, and endocardium. Moreover, the authors elegantly proved that the three-lineage-model of hCOs responds to a cardiac injury similarly to the human heart tissue by triggering the migration of fibroblast-like cells toward the injured site and promoting the local accumulation of ECM (Hofbauer et al., 2021).

A pioneering study by Lewis-Israeli and others increased the structural complexity of hCOs by applying a three-step Wnt signaling modulation protocol (activation/inhibition/activation) (Lewis-Israeli et al., 2021b). Indeed, the second exogenous induction of the Wnt pathway promotes the clustering of epicardial cells in the external layer of the hCOs, ultimately surrounding the myocardial tissue (Figure 1E). Interestingly, the authors showed that it is possible to model pregestational diabetes-induced congenital heart disease “in a dish” by finely tuning hCO exposure to glucose and insulin concentrations mimicking the pathological diabetic values. Strikingly, this treatment led to the formation of hCOs carrying structural defects and altered metabolism (Lewis-Israeli et al., 2021b).

Drakhlis et al. generated hCOs by encapsulating hPSC aggregates in Matrigel droplets that are subsequently differentiated using a modified GiWi protocol (Drakhlis et al., 2021a). The authors referred to these organoids as “Heart-Forming Organoids” (HFOs). HFOs have a complex organization characterized by an inner and outer layer, patterning anterior and posterior foregut endoderm, respectively. These structures are separated by a ring-shaped mid-layer which resembles the primitive streak specification and the subsequent cardiac *primordium* (Drakhlis et al., 2021a; Drakhlis et al., 2021b) (Figure 1F). Furthermore, the potential of the HFOs as an *in vitro* model of early cardiogenesis was tested by generating NKX2.5-KO HFOs. Surprisingly, NKX2.5-KO HFOs displayed loosely arranged CMs in the mid-layer and larger diameter than *wild-type* HFOs (Drakhlis et al., 2021a). Notably, the phenotype of NKX2.5-KO HFOs is reminiscent of the lack of tissue compactness observed in NKX2.5-KO transgenic mice (Lyons et al., 1995).

### 3D-Derived Cardiac Microchambers

The controlled biophysical features of micropatterning allow studying differentiation more in detail than traditional methods based on the formation of heterogeneous embryoid bodies (Warmflash et al., 2014). Therefore, scientists developed strategies for generating hCOs in geometric-confined microchambers (Ma et al., 2015). To evaluate the influence of the geometric confinement on cardiac differentiation, scientists fabricated microchambers of circular, triangular, and squared cell culture areas at the micron scale using standard lithography techniques and oxygen plasma etching of tissue culture plates (Ma et al., 2015; Hoang et al., 2018). hPSCs were seeded in Matrigel-coated microchambers and differentiated employing the GiWi protocol. These geometric confinements produced a beating CM area at the center and an increased myofibroblast density at the perimeter of the microchamber (Ma et al., 2015). This model was used to evaluate the developmental toxicity of nine drugs with different reported toxicity grades during pregnancy. Interestingly, most of the tested drugs resulted in abnormal CM contraction and impaired hCO formation. Hoang and others tested the same nine drugs *in vivo* on Zebrafish whole embryos and confirmed a developmental toxicity comparable to the *in vitro* hCO model (Hoang et al., 2021).

## Directed-Assembly Method

### Engineered Heart Tissues

The directed-assembly method produces a type of hCOs acquiring heart tissue-like structures, also known as Engineered Heart Tissues (EHTs). Overall, this methodology combines hPSC derived-cardiac cells and ECM substrates in casting molds to generate hydrogels (Figure 1G). These cell-laden bioengineered hydrogels require a few days to condensate and acquire their final scaffold properties (Zimmerman et al., 2000; Mills et al., 2017; Tiburcy et al., 2017; Vogues et al., 2017; Ronaldson-Buochard et al., 2018; Mills et al., 2019; Zhao et al., 2019; Mills et al., 2021). Nowadays, scientists have developed various methodologies for deriving shape-controlled EHTs that differ for differentiation stimuli, cell subtypes, and ECM composition.

Tiburcy and others recently improved CM maturation in EHTs and defined the cellular and chemical components to fulfill the good manufacturing practice required for clinical translation (Tiburcy et al., 2017). hPSC derived-CMs and human foreskin fibroblasts were cast in medical-grade type I collagen hydrogels to form ring-shaped EHTs that were mechanically stimulated by dynamic stretching. CMs isolated from these EHTs showed rod-shaped and a remarkable degree of sarcomere organization, besides the postnatally developed M-bands (Kamakura et al., 2013). Additionally, mature EHTs showed enhanced contractile force in response to increased pacing frequencies (Tiburcy et al., 2017), a human heart trait developed along the first year after birth (Wiegerinck et al., 2009). Moreover, EHTs responded inotropically (by enhancing the force) and lusitropically (by increasing relaxation) to *β*-adrenergic stimulation. Furthermore, chronic catecholamine-treated EHTs resemble a heart failure model, including contractile dysfunction, CM hypertrophy, and Natriuretic Peptide release (Yang and Murry, 2017). Although Tiburcy’s EHTs improved several CM maturation features compared to previous methods, their clinical application is hindered by substantial differences with the adult human heart. These EHTs have a lower force of contraction and lower CM volume than the adult heart, and their transcriptome recapitulates the expression profile of the human fetal heart (Tiburcy et al., 2017, Yang and Murry, 2017).

Another approach to promote CM maturation employs electromechanical stimulation (Ronaldson-Bouchard et al., 2018). This method combines hPSC-derived CMs at the early stage of differentiation and human dermal fibroblast in a 3:1 ratio. The cell mixture is encapsulated in a fibrin hydrogel cast around two elastomeric pillars designed to mimic the mechanical load of the native human myocardium. After several days in culture, hCOs were subjected to intensive training by modulating the electrical stimulation. The intensive training enhanced the maturation of CMs that displayed highly organized sarcomeres, gap junctions at CM poles, and a remarkable transverse (T)-tubule network. Despite the maturation hallmarks attained by intensively trained CMs, the native human myocardium is characterized by higher magnitudes of contraction, action potential upstroke, and conduction velocity (Hasenfuss et al., 1991).

Both Tiburcy’s and Ronaldson-Bouchard’s approaches harbored a degree of complexity and required expertise that have potentially limited their usage and throughput. On the contrary, the Heart dynamometer (Heart-dyno), developed by Mills et al. in light of the minimal required tissue handling, has been constantly used in recent years. This method relies on a 96-well plate screening platform to identify favorable conditions for hCO growth, maturation, and functions (Mills et al., 2017; Mills et al., 2019; Mills et al., 2021). This approach generates an hPSC-derived cardiac cell population composed of 70% CMs and 30% mural cells, subsequently mixed with collagen I and Matrigel to cast a hydrogel in a cell culture insert-well containing two elastomeric poles. Like previous approaches, the hydrogel condensates around the poles during 5 days forming the hCOs, which are exposed to mechanical load by the elasticity of the poles for ten additional days. Using the Heart-dyno, the authors identified a chemically defined maturation media supplemented with palmitate, reduced glucose, and no insulin. These conditions improved contraction forces and expression of ventricular Myosin regulatory Light Chain 2 (*MLC2v*), decreased cell cycle activity, and induced a metabolic switch from glycolysis to fatty acid oxidation, which increased CMs oxygen consumption rate and mitochondrial content (Mills et al., 2017).

## Application of Human Cardiac Organoids as a Drug Screening Platform

The directed assembly method promotes the maturation of CMs by mechanical load and electrical stimulation. The enhanced maturation of CMs made this approach suitable for drug screening studies, testing CM pro-proliferative compounds, and modeling SARS-CoV-2-induced cardiac complications *in vitro* (Mills et al., 2017; Voges et al., 2017; Mills et al., 2019; Mills et al., 2021).

The Heart-dyno system has been recently used to investigate the bilateral ventricular diastolic dysfunctions developed in SARS-CoV-2 patients (Mills et al., 2021). To identify the pro-inflammatory cytokines driving the cardiac dysfunction, also known as cardiac cytokine storm, Mills and others screened several pro-inflammatory cytokines elevated in SARS-CoV-2 patients on hCOs. The combination of IFN-*γ*, IL-1*β*, and poly(I:C) caused a robust diastolic dysfunction together with bromodomain-containing protein 4 (BRD4) activation. Significantly, treatment with bromodomain 2 (BD2)-selective BET inhibitors reduced the cytokine storm-induced diastolic dysfunction in hCOs and SARS-CoV-2 infected mice, and has the potential to reduce SARS-CoV-2 infection of cardiac cells (Mills et al., 2021).

## Limitation and Future Perspective

Here, we reviewed some advanced techniques for the production of cardiac 3D organoids starting from hPSCs. The approaches described above generate morphological and functional models that partially resemble the *in vivo* heart development. Importantly, each model displays unique characteristics that suit specific purposes, but all show some limitations.

ULA-derived self-assembled hCOs represent helpful *in vitro* models to recapitulate the early stages of heart development and the dynamic cell-to-cell interactions occurring during cardiomyogenesis. This method relies on the intrinsic capacity of hPSCs to self-organize, implying a stochastic arrangement in the three-dimensional space. Thus, this variability hampers the reproducibility of the studies (Jabaudon and Lancaster, 2018). On the other hand, the micropatterning platform represents a valuable alternative to develop spatially organized multicellular structures. However, the contribution of batch-to-batch variable components of Matrigel along the differentiation should be carefully evaluated (Pagliarosi et al., 2020) and substituted with well-defined synthetic molecules whenever possible (Gjorevski et al., 2016). Unfortunately, the structural complexity of *in vivo* organs, such as the four cardiac chambers, valves, and inflow/outflow tract, cannot be faithfully recreated with these models. Furthermore, self-assembly-derived hCOs resemble the characteristics of fetal-like organs and cannot be employed to model heart diseases occurring in later stages of development. In this regard, the directed assembly approach offers a promising alternative based on casting platforms, such as the EHTs, that provide an excellent cellular system to optimize CM maturation while more closely mimicking the *in vivo* physiology. However, EHT-derived CMs fail to fully recapitulate the adult heart tissue anatomy. Interestingly, the recent application of the Heart-dyno model in SARS-CoV-2 research showed its tremendous potential. Innovative approaches could be based on the formation of multicellular “assembloids,” as recently reported for the brain tissue (Paşca, 2018). Assembloids were first reported by Paşca’s group that fused cortical and subpallium spheroids, reproducing an unprecedented neural circuit (Birey et al., 2017). This approach gave rise to interregional interactions that had never been investigated before. Thus, we foresee a possible application of this technique to generate heart assembloids that include cardiac-related cell lineages.

In conclusion, we reviewed the latest 3D methodologies to derive hCOs from hPSCs, which promise to further advance translational medicine applications of hPSCs. The use of these models could provide novel insights into understanding human cardiogenesis, injury processes, and developmental drug toxicities.
